# Artificial Synapse Consisted of TiSbTe/SiC_x_:H Memristor with Ultra-high Uniformity for Neuromorphic Computing

**DOI:** 10.3390/nano12122110

**Published:** 2022-06-19

**Authors:** Liangliang Chen, Zhongyuan Ma, Kangmin Leng, Tong Chen, Hongsheng Hu, Yang Yang, Wei Li, Jun Xu, Ling Xu, Kunji Chen

**Affiliations:** 1 School of Electronic Science and Engineering, Nanjing University, Nanjing 210093, China; puppymoon@163.com (L.C.); lengkangmin@foxmail.com (K.L.); chentong1110@foxmail.com (T.C.); xiaohuyz@163.com (H.H.); 2185223301@qq.com (Y.Y.); weili@nju.edu.cn (W.L.); junxu@nju.edu.cn (J.X.); lingxu@nju.edu.cn (L.X.); kjchen@nju.edu.cn (K.C.); 2 Collaborative Innovation Center of Advanced Microstructures, Nanjing University, Nanjing 210093, China; 3 Jiangsu Provincial Key Laboratory of Photonic and Electronic Materials Sciences and Technology, Nanjing University, Nanjing 210093, China

**Keywords:** memristor, uniformity, stabilization

## Abstract

To enable a-SiC_x_:H-based memristors to be integrated into brain-inspired chips, and to efficiently deal with the massive and diverse data, high switching uniformity of the a-SiC_0.11_:H memristor is urgently needed. In this study, we introduced a TiSbTe layer into an a-SiC_0.11_:H memristor, and successfully observed the ultra-high uniformity of the TiSbTe/a-SiC_0.11_:H memristor device. Compared with the a-SiC_0.11_:H memristor, the cycle-to-cycle coefficient of variation in the high resistance state and the low resistance state of TiSbTe/a-SiC_0.11_:H memristors was reduced by 92.5% and 66.4%, respectively. Moreover, the device-to-device coefficient of variation in the high resistance state and the low resistance state of TiSbTe/a-SiC_0.11_:H memristors decreased by 93.6% and 86.3%, respectively. A high-resolution transmission electron microscope revealed that a permanent TiSbTe nanocrystalline conductive nanofilament was formed in the TiSbTe layer during the DC sweeping process. The localized electric field of the TiSbTe nanocrystalline was beneficial for confining the position of the conductive filaments in the a-SiC_0.11_:H film, which contributed to improving the uniformity of the device. The temperature-dependent *I*-*V* characteristic further confirmed that the bridge and rupture of the Si dangling bond nanopathway was responsible for the resistive switching of the TiSbTe/a-SiC_0.11_:H device. The ultra-high uniformity of the TiSbTe/a-SiC_0.11_:H device ensured the successful implementation of biosynaptic functions such as spike-duration-dependent plasticity, long-term potentiation, long-term depression, and spike-timing-dependent plasticity. Furthermore, visual learning capability could be simulated through changing the conductance of the TiSbTe/a-SiC_0.11_:H device. Our discovery of the ultra-high uniformity of TiSbTe/a-SiC_0.11_:H memristor devices provides an avenue for their integration into the next generation of AI chips.

## 1. Introduction

Brain-inspired neuromorphic computing is attracting more and more attention as a new computing system that has the potential to overcome the limitations of traditional von Neumann computational architecture [1,2]. Therefore, artificial synaptic devices for brain-inspired neuromorphic computing have been increasingly studied in recent years. As with the two-terminal structure of the biological synapse, resistive random-access memory (RRAM), also a two-terminal device, is considered to be the most promising candidate for electronic synaptic devices, due to its similarity to a characteristic of the biological synapse [2,3], whose conductance can be modified dynamically by changes in both ion and electron current flow [4]. A large amount of fascinating work has been performed to develop oxide-based RRAMs for neuromorphic systems, with promising results, such as HfOx-based synaptic devices [5], TiO_x_-based synaptic devices [6], Ag/STO:Ag/SiO_2_/p++-Si structure [1], and TiN/SiO_x_/TiN [4] structure. Among these, silicon-based materials are highly valued, due to their compelling advantage of being fully CMOS-compatible [7], which can greatly simplify the circuit. Zarudnyi et al. [4] demonstrated the spike-timing-dependent plasticity of silicon-rich silicon oxide (SiO_x_), which confirmed that SiOx-based RRAM is a good biological synapse device. Furthermore, SiO_x_ switching layers ensure full compatibility with complementary metal-oxide semiconductor (CMOS) technology, leading to a reduction in circuit complexity. However, the set voltage of SiO_x_-based devices fluctuates in the range of 3.5 V to 4 V, and the reset voltage is around 2.5 V, showing relatively high power consumption. Furthermore, the endurance test of 150 cycles showed a large fluctuation in the LRS, which indicates that the uniformity of the Si-based device still needs to be improved.

With the approaching era of big data and artificial intelligence, a next-generation memristor with high uniformity, high density, and low consumption is urgently needed, to construct the artificial synapse, which is the key to ensuring the realization of the brain-inspired chip [8,9,10,11]. Compared with SiO_x_-based RRAM, SiC-based resistive switching memory has recently attracted great interest due to its promising advantages, such as tunable conductance, which is beneficial for obtaining controllable synaptic weight. In particular, SiC-based resistive memory with a forming-free characteristic can be easily integrated into the neuromorphic computing chip, with a simple peripheral circuit at a low cost [12,13,14]. To enable the SiC-based memristor to integrate into the brain-inspired chip and efficiently deal with the massive and diverse data, the uniformity of the switching parameters such as R_ON_, R_OFF,_ V_SET_, and V_RESET_ need to be improved in the successive switching cycles. Exploring effective ways to improve the switching uniformity of the SiC memristor is highly demanded [15,16,17,18,19,20,21,22,23,24].

In this study, we report an efficient way to obtain the ultra-high uniformity of switching parameters by introducing a TiSbTe (TST) layer into the top electrode and the a-SiC_0.11_:H. In our previous work [25], we obtained three kinds of a-SiC_x_:H-based devices, with C/Si ratios of 0.11, 0.25, and 0.49, by tuning SiH4/CH4 flow ratios of 5/1, 6.25/1, and 10/1, respectively. We found that the memory window increased from 10 to 10^3^, with the C/Si ratio decreasing from 0.49 to 0.11. The Al/a-SiC_0.11_:H/Si device had the smallest set voltage and the best stability among the three devices. Thus, we chose to conduct further research on the Al/a-SiC_0.11_:H/Si device. We found that, compared to the Al/a-SiC_0.11_:H/Si^+^ (ASS) memristor, the cycle-to-cycle coefficient of variation in the high resistance state and the low resistance state of the Al/TiSbTe/a-SiC_0.11_:H/Si^+^ (ATSS) memristor could be reduced by 92.5% and 66.4%. Moreover, the device-to-device coefficient of variation in the high resistance state and the low resistance state of the Al/TiSbTe/a-SiC_0.11_:H/Si^+^ memristor could be reduced by 93.6% and 86.3%. HRTEM revealed that the significantly improved uniformity could be attributed to the confinement role of the TST crystalline nanopathway, which combined with the Si dangling bond to form a stable, conductive pathway. The conduction mechanism of the Al/TST/a-SiC_0.11_:H/Si^+^ devices in both the LRS and the HRS was further analyzed through temperature-dependent current analyses.

## 2. Materials and Methods

**Figure 1:**
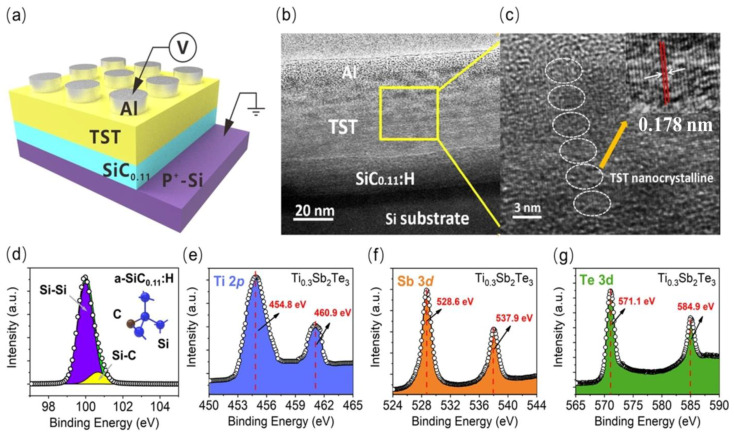
(**a**) The schematic diagram of the Al/TST/a-SiC_0.11_:H/P^+^-Si memory device after the DC sweeping electrical measurement; (**b**) Cross-section TEM of Al/TST/a-SiC_0.11_:H/P^+^-Si RRAM device after the first setting process; (**c**) High-resolution cross-section TEM of TST nanocrystalline formed in the Al/TST/a-SiC_0.11_:H/P^+^-Si RRAM device after electric measurement; The TST nanocrystalline dot is marked by the circles. And the inset shows the interplanar spacing of TST nanocrystalline is 0.178 nm, which is marked by the distance of two red lines. (**d**–**g**) XPS spectra of a-SiC_0.11_:H and Ti 2*p*, Sb 3*d*, Te 3*d* in Ti_0.3_Sb_2_Te_3_.

Figure 1a,b show the structural diagrams of the Al/TST/a-SiC_0.11_:H/P^+^-Si memory device. An a-SiC_0.11_:H film of 30 nm thickness was deposited on the P^+^-Si substrate by a plasma-enhanced chemical vapor deposition system at 250 °C. The gas precursors consisted of SiH_4_ and CH_4_, with a volume ratio of 5:1. Then, a 30 nm-thick TST layer was grown on the surface of the a-SiC_0.11_:H films, using an ACS-4000-cs magnetron sputtering system at room temperature. The power of the magnetron sputtering was 50 W. For comparison, a reference device of 30 nm thickness, without a TST layer, was also fabricated in parallel. Finally, aluminum top electrodes, with differing diameters, were deposited via thermal evaporation. The top electrodes of our device were circular aluminum, with diameters of 1000 nm, 800 nm, and 500 nm. The corresponding device area was 0.79 um^2^, 0.50 um^2^, and 0.20 um^2^. Aluminum was also deposited at the backside of the Si substrate as the back electrode, for better contact. The atomic concentration ratios of the a-SiC_0.11_:H films and the TST films were obtained from the surface of the corresponding films by an XPS test, using the PHI 5000 Versa Probe (Ulvac-Phi Inc., Chigasaki, Japan). The microstructures of the Al/TST/a-SiC_0.11_:H/P^+^-Si memory device after electric measurement were analyzed using high-resolution cross-section transmission electron microscopy (HRTEM) (JEOL Inc., Tokyo, Japan), with a JEOL 2100F electron microscope operated at 200 kV. Electrical characterizations of the memory devices were performed using an Agilent B1500A semiconductor analyzer (Agilent Inc., Santa Clara, CA, USA). The CRX-4K Lake Shore system was adopted to analyze the temperature-dependent *I*-*V* characteristic under a vacuum of 5 × 10^−5^ Torr (Lake Shore Inc., Westerville, Ohio, USA).

## 3. Results and Discussion

A cross-sectional TEM image of the Al/TST/a-SiC_0.11_:H/P^+^-Si RRAM device after electric measurement is displayed in Figure 1b. Clear interface of TST and a-SiC_0.11_:H was identified, revealing that the thicknesses of the TST layer and the a-SiC_0.11_:H layer were 40 and 30 nm, respectively. As shown in Figure 1c, the TST nanocrystalline could be seen clearly to be distributed in the TST layer, which formed a continuous nanopathway in the Al/TST/a-SiC_0.11_:H/P^+^-Si RRAM device. Interestingly, the TST nanocrystalline could not switch from its crystalline state to an amorphous state in the following negative or positive DC sweeping. Usually, to obtain phase changing of TST materials, a short electrical pulse is used to achieve the amorphous state (the high resistance RESET state); a lower, but slightly longer current pulse, is needed to convert it to the polycrystalline state (the lower resistance SET state) [26]. Here, the duration of the DC voltage in our set, and the reset progress, was ms, which could produce enough Joul heat to make the TST films transform from the amorphous state to the crystalline state. Therefore, the TST dot remained in the crystalline state, which ensured that the TST nanopathway could not be ruptured in the following cycles. The composition analysis of the TST/a-SiC_0.11_:H films was conducted by using X-ray photoelectron spectroscopy. Figure 1d shows the Si 2*p* spectrum of the a-SiC_0.11_:H, which was deconvoluted into two Gaussian peaks related to Si–Si (99.2 eV) and Si–C (100.8 eV). Figure 1e–g display the Ti 2*p*, Sb 3*d*, and Te 3*d* peaks from the surface of the Ti_0.3_Sb_2_Te_3_ film. The peaks corresponding to Sb 3*d* are located at 528.6 eV and 537.9 eV, while the peaks related to Te 3*d* are centered at 571.1 eV and 584.9 eV. Compared to Sb_2_Te_3_, the peaks of Te-3*d* and Sb-3*d* have lower energy with Ti doping, which indicates that the original bonding structure changed. The doped Ti atoms likely replaced the Sb, and formed Ti-Te bonds, and a stable structure of TST was thereby formed.

**Figure 2:**
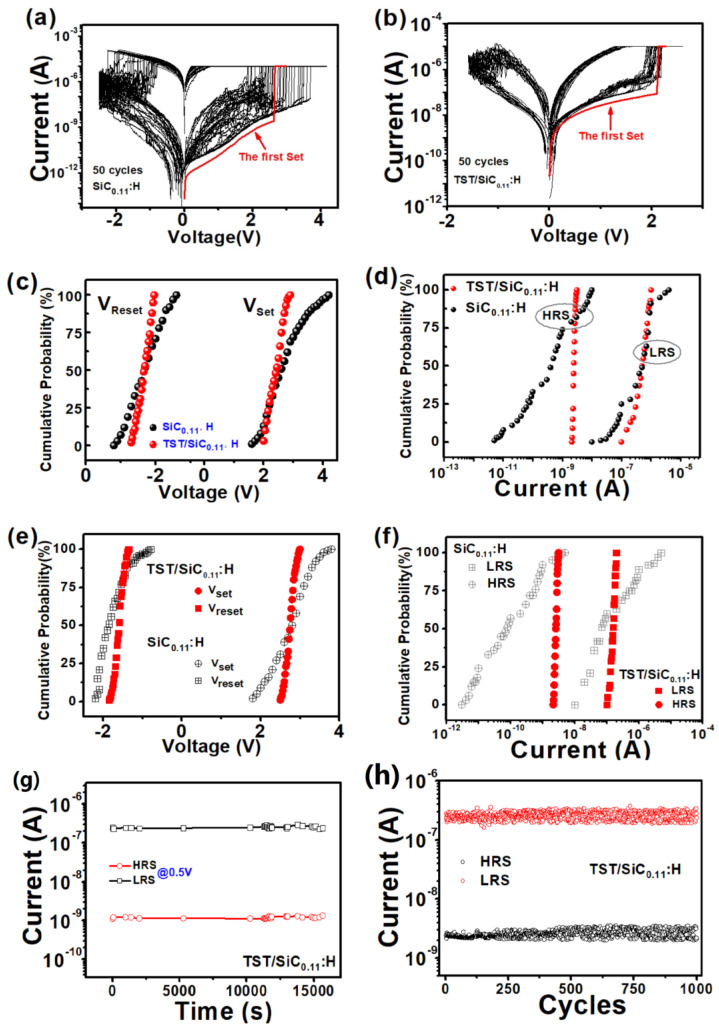
(**a**,**b**) Resistive switching I–V curves of ASS device and ATSS device during the SET and RESET process after 50 cycles as marked by black lines and the first set is marked by red lines. (**c,d**) Accumulative probability of Vset, Vreset, Ron, and Roff after 50 cycles; (**e**,**f**) Variation distribution of device-to-device Vset, Vreset, and LRS/HRS values. Data were obtained from 30 ASS and ATSS memristor devices; (**g**) Retention characteristics of the ATSS memristor device at 80 °C; (**h**) The endurance characteristic of the ATSS memristor.

To compare the performances of the ASS and the ATSS memristor devices, current voltage (I–V) curves of 50 consecutive cycles are shown in Figure 2a,b, respectively. During the electrical measurement, a 10-uA compliance current was used to protect the devices from permanent breakdown. The red lines in the inset represent their first sweep. It is worth noting that both samples exhibited forming-free behavior, as the first sweeping voltage was within the range of the following set voltages. An interesting finding was that the first sweeping voltage of the ATSS devices was 2.2 V, which was lower than the 2.7 V of the ASS device. This means that the conductivity of the nanopathway was enhanced after the introduction of the TST layer on to the ASS device, which was related to the appearance of the TST nanocrystalline. Meanwhile, the ATSS devices exhibited highly improved uniformity compared with that of the ASS devices. During the 50 cycles, the V_set_ and V_reset_ range of the ASS were 1.8~3.8 V and −0.8~−2.3 V, while those of the ATSS were 1.9~2.25 V and −1.0~−1.5 V, respectively. Evidently, the dispersion of the switching curves was greatly minimized in the ATSS structure. Figure 2c,d shows the accumulative probability of V_set_ and V_reset_, R_on,_ and R_off_, during the 50 set/reset switching cycles under DC voltage. It was evident that the fluctuation of the V_set_/V_reset_, and the HRS/LRS of the ATSS devices, were sharply reduced, in contrast to that of the ASS device. Compared with that of the a-SiC_0.11_:H memristor, the cycle-to-cycle coefficient of variation in the high resistance state and the low resistance state of the TiSbTe/a-SiC_0.11_:H memristor was reduced by 92.5% and 66.4%, respectively. In addition, the variation of device-to-device parameters in these two types of device is displayed in Figure 2e,f: they were detected in 30 random devices after 30 cycles. The accumulative probability of the V_set_/V_reset_ and the HRS/LRS values showed that the ATSS device had a narrower distribution of device-to-device parameters, further confirming the remarkable uniformity. Compared to that of the a-SiC_0.11_:H memristor, the device-to-device coefficient of variation in the high resistance state and the low resistance state of the 30 randomly selected TiSbTe/a-SiC_0.11_:H memristors was reduced by 93.6% and 86.3%. The retention and the endurance characteristics of the TiSbTe/a-SiC_0.11_:H memristor are presented in Figure 2g,h, respectively. No degradation was observed from the two resistance states at 80 °C after 10^4^ s (read at 0.5 V), as shown in Figure 2g. The memory window of 10^2^ also remained stable. In addition, the reproducibility of the device was also displayed by the repetition of the RS behavior for 1000 cycles, as shown in Figure 2h. The ATSS device clearly maintained high stability, with no misreading of resistance states or switching failure during the measurement, which demonstrated high resistive switching reliability.

**Figure 3:**
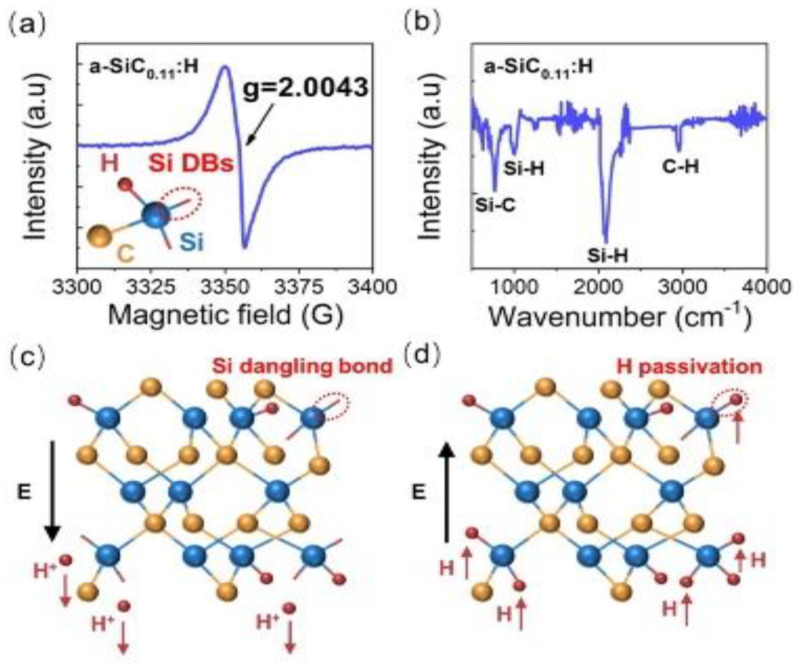
(**a**,**b**) ESR and FTIR spectra of as-deposited a−SiC_0.11_H films; (**c**) Under a positive bias, Si dangling bonds were formed, due to the broken Si–H bonds; (**d**) Under a negative bias, Si dangling bonds were passivated by H^+^.

In order to understand the correlation between resistive switching and the atomic configurations of a-SiC_0.11_H films, the corresponding Fourier transform infrared (FTIR) spectroscopy and electron spin resonance (ESR) spectra were analyzed, as shown in Figure 3a,b. A resonance peak with a g value of 2.0043 can be observed for the a-SiC_0.11_:H films, which was induced by the Si dangling bonds (Si DBs) in the a-SiC_x_. This indicates that Si DBs were formed in the a-SiC_0.11_:H films during the chemical vapor deposition. As shown in Figure 3b, FTIR peaks related to the Si-H wagging vibrations, the Si-H stretching vibrations, the Si-C stretching vibrations, and the C-H stretching modes, could be detected in absorption bands of 1000 cm^−1^, 2000 cm^−1^, 780 cm^−1^, and 2900 cm^−1^, respectively. The C-H bonds and Si-H bonds were derived from the hydrogenation of the silicon carbon. The higher intensity of both the Si-H wagging mode and the Si-H stretching mode indicated that there were a number of Si-H bonds in the as-deposited films. The above results indicated that there were Si DBs and Si-H bonds in the a-SiC_0.11_:H films in their initial state. As the Si-C bond energy (3.7 eV) and the C-H bond energy (4.2 eV) was higher than that of the Si-H bond (3.0 eV), the Si-H bonds were easier to break, which generated Si dangling bonds under the electric field. As displayed in Figure 3c, Si DBs were produced, as the Si-H bonds could be broken under the positive electric field, and hydrogen ions migrated towards the negative cathode. When the number of Si DBs reached a high level, a filament of Si dangling bonds was formed, causing the device to switch from an HRS to an LRS. Under negative bias, the migration of H^+^ ions was able to passivate some Si DBs, causing the Si dangling bond conductive pathway to be disconnected. Thus, the device could be switched back to an HRS.

**Figure 4:**
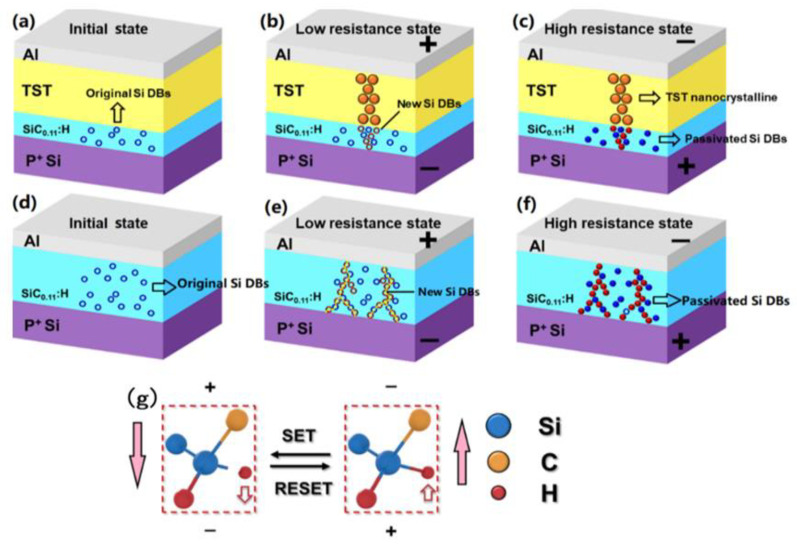
The resistive switching model of the Al/TST/a-SiC_0.11_:H/P^+^-Si device: (**a**) In the initial state, the Si dangling bonds distribute in the a-SiC_0.11_:H layer, which are represented by the blue circle; (**b**) In the LRS, TST nanocrystalline was produced under the DC positive voltage. The localized electric field of the TST nanocrystalline induced more Si DBs to aggregate under the TST nanocrystalline, forming an Si DB-conductive nanopathway; (**c**) In the HRS, H^+^ moved back to accurately passivate the original Si dangling bonds under negative voltage, resulting in the rupture of the conductive nanopathway; (**d**) The resistive switching model of the Al/a-SiC_0.11_:H/P^+^-Si device. In the initial state, the Si dangling bonds were distributed in the a-SiC_0.11_:H layer, and are represented by the blue circles; (**e**) In the LRS, new Si DBs were formed from broken Si-H bond under a positive bias, forming a conductive nanopathway; (**f**) In the HRS, H^+^ moved back to passivate some Si dangling bonds under a negative voltage, resulting in the rupture of the conductive nanopathway; (**g**) The breakage of the Si-H bonds and the passivation of the Si dangling bonds by H^+^ under the electric field direction of forward and reverse direction made the pathway be broken up and connected.

To explore the reason for the suppression of the dispersion of the switching parameters after the introduction of the TST layer into the ASS device, a confined resistive switching nanopathway model of the ATSS device is illustrated in Figure 4. In the initial state (I_S_), a number of Si DBs pre-existed randomly in the a-SiC_0.11_:H layer, as shown in Figure 4a. Under a positive voltage, the TST layer transformed from an amorphous state to a crystalline state, due to the heat of the increased current, generating a permanent TST nanofilament in the TST film, which was confirmed by the HRTEM photograph in Figure 1c. Meanwhile, more Si-H bonds were able to aggregate near the tip of the TST nanopathway, causing it to break, generating new Si dangling bonds in the a-SiC_0.11_:H layer under the localized electric field [15,27,28]. When the number of Si DBs increased to a certain extent, they formed a conductive pathway and were connected with the TST nanopathway, switching the device to an LRS, as illustrated in Figure 4b. When negative voltage was applied, those Si DBs in the original nanopathway were accurately passivated by H^+^, due to the role of the localized electric field induced by the TST nanocrystalline. The passivation of the Si dangling bonds resulted in the rupture of the conductive nanopathway, as displayed in Figure 4c. Therefore, the device was able to return to an HRS. Here, the TST nanopathway supplied a “fixed position” to confine the growth and rupture of the Si dangling bonds during the RS process. In contrast to the AST device, the distribution of the Si DB nanopathway under a positive bias was random in the ASS device, without the confinement of the TST nanopathway as shown in Figure 4e. Under a negative bias, not all Si dangling bonds in the original nanopathway were accurately passivated by H^+^, which resulted in the fluctuation of the switching parameters during the successive cycles [29]. It was evident that the TST nanocrystalline was beneficial for improving the uniformity of the conductive nanopathway guidance, while the formation and passivation of the Si dangling bond were responsible for the bridge and rupture of the conductive nanopathway. As displayed in Figure 4g, new Si DBs were produced from the broken Si-H bonds under the positive electric field. When the bias changed to negative, the Si DBs were passivated by H^+^. Therefore, it can be seen that a combined conductive nanopathway of TST nanocrystalline and Si DBs can improve the uniformity of the switching parameters of the ATSS device.

**Figure 5:**
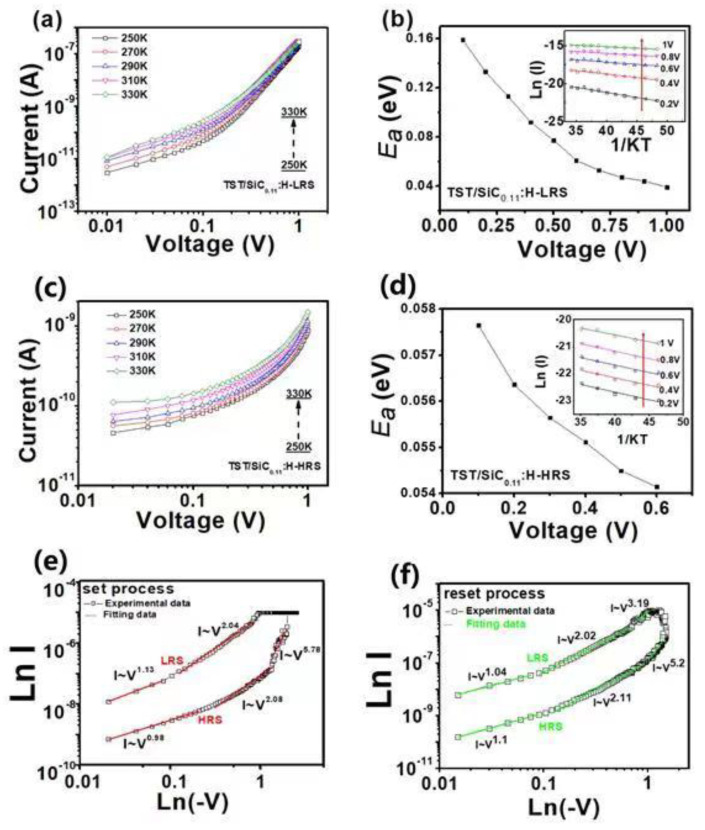
(**a**–**c**) Temperature-dependent current of the ATSS device in the HRS and the LRS at temperatures from 250 K to 330 K; (**b**–**d**) The activation energy of the ATSS device in the HRS and LRS, which is decreased with the voltage increasing. The inset shows an Arrhenius plot of the temperature-dependent current of the HRS and the LRS. The current intensity is enhanced with the voltage increasing as marked by the red arrows; (**e**,**f**) The experimental and theoretical fitting curves of the set and reset process of the Al/a-SiC_0.11_:H/p^+^Si device.

To further illustrate the role of the Si dangling bond conductive nanopathway on the resistive switching process of the Al/TST/a-SiC_0.11_:H/P^+^-Si RRAM, the temperature dependence of the HRS and the LRS currents is shown in Figure 5a–c. It can be seen that the current values of the LRS and the HRS increased as the temperature increased from 250 to 330 K, indicating a semiconductor-like conduction behavior [28,29,30]. According to the slopes of Arrhenius-type plots, the activation energy of the HRS and the LRS was obtained in Figure 5b,d. In the LRS, the E_α_ was reduced from 0.15 eV to 0.04 eV, with the voltage increasing from 0.125 to 1 V. The same changing trend of Ea was observed in the HRS, which decreased from 0.058 to 054 eV, with the voltage increasing from 0.1 to 0.6 V. Both showed characteristic SCLC conduction behavior [31]. The I-V characteristic curves of the HRS and the LRS were replotted in double logarithmic scales for illustrating the set process and reset process in the Al/TST/a-SiC_0.11_:H/P^+^-Si device. As shown in Figure 5e, hot mobile electrons made the contribution to the carrier transformation via thermal excitation in the conduction band, and followed the Ohmic conduction rule, with an I–V slope of 0.98 under low voltage during the set process. With the bias increasing, the Si-H bond was broken, to form Si DBs under the electric field, with H^+^ ions migrating towards the negative cathode. With the number of the Si DBs increasing, a conductive nanopathway was formed, causing the current to deviate from Ohm’s law to Child’s law, with an *I*–*V* slope of 2.08. When the voltage (Vset) was increased to a higher level, the number of Si DBs reached maximum. A remarkable enhancement of the current was observed, with the slope increasing from 2.08 to 5.78, switching the memory device from an HRS to an LRS. In the initial low resistance region, with a slope of 2.04, when the device was still in the LRS state, all Si DBs remained filled. However, the number of the carrier injection reduced with the decreased voltage. Therefore, the slope decreased from 2.04 to 1.13 in the low-voltage region, revealing that current conduction behavior in the LRS was consistent with the SCLC mechanism. The Ln (I) versus Ln (-V) curves of the reset process are also shown in Figure 5f. Before the voltage reached the Vreset, the occupied Si DBs made the device keep to an LRS. Once the bias increased to Vreset, the Si DBs began to discharge electrons in the high-voltage region. Meanwhile, more and more Si DBs were passivated by H^+^ ions under negative voltage. Thus, electrons were released from most of the occupied Si DBs, accompanied by the rupture of the Si DB conductive pathway, switching the memory device from an LRS to an HRS. Therefore, both the LRS and the HRS could be dominated by the Si DBs-controlled SCLC mechanism, which was consistent with the results of the temperature-dependence analysis.

**Figure 6:**
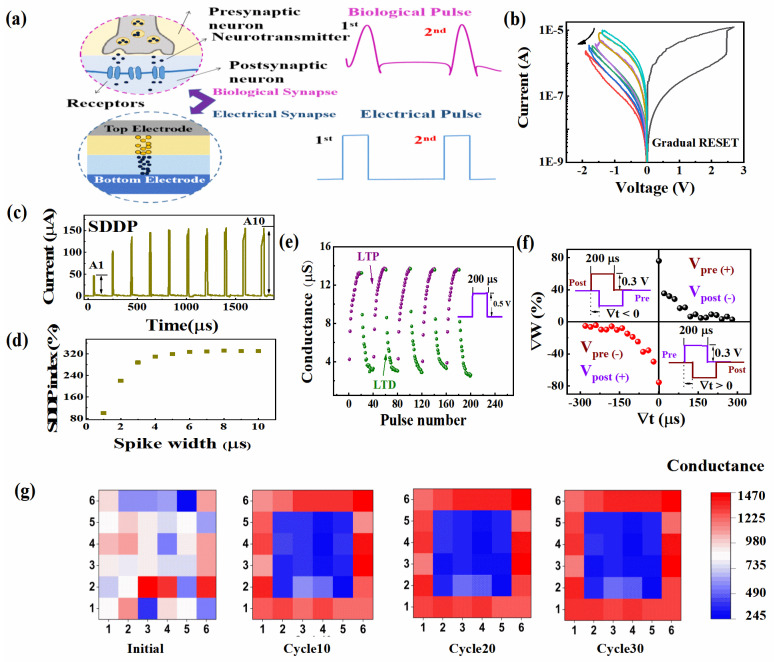
(**a**) A schematic diagram of biological synapse and electrical synapse; (**b**) Multilevel resistive switching characteristics of the Al/TiSbTe/a-SiC_0.11_:H/P^+^-Si device, with different reset stop voltage; (**c**) The SDDP characteristics of the Al/TiSbTe/a-SiC_0.11_:H/P^+^-Si device; (**d**) The SDDP index (An/A1∙100%; *n* = 1, 2, 3, …, 10) of the Al/TiSbTe/a-SiC_0.11_:H/P^+^-Si device; (**e**) Incremental conductance changes through 20 positive pulses (0.5 V, 200 µs) followed by 20 negative pulses (−0.5 V, 200 µs); (**f**) Spike-timing-dependent plasticity (STDP) of the Al/TiSbTe/a-SiC_0.11_:H/P^+^-Si device through the overlapping of the pre-synaptic spike and the post-synaptic spike; (**g**) The simulation of image memorization under consecutive electrical pulses.

To enable the Al/TiSbTe/a-SiC_0.11_:H/P^+^-Si device to be used as an artificial synapse for integration into a brain-inspired chip, we studied the long-term memory and the short-term memory characteristics of the Al/TiSbTe/a-SiC_0.11_:H/P^+^-Si artificial synapse. As shown in Figure 6a, the structure of the biological synapse was similar to that of the prepared Al/TiSbTe/a-SiC_0.11_:H/P^+^-Si device. The pre-synaptic and post-synaptic neurons could be regarded as the top electrode and the bottom electrode, respectively. To make the Al/TiSbTe/a-SiC_0.11_:H/P^+^-Si device emulate the function of the biosynapse, the multilevel resistive switching characteristics of the Al/TiSbTe/SiC_0.11_:H/P^+^-Si device were measured by controlling the reset stop voltages, as shown in Figure 6b. The current decreased gradually with the increasing of negative voltage, which was similar to the tunable synaptic weight. We studied the short-term characteristic of the TiSbTe/SiC_0.11_:H artificial synapse, which was spike-duration-dependent plasticistic (SDDP). The synaptic weight of the TiSbTe/SiC_0.11_:H synapse was effectively amplified by prolonging the duration of the synaptic spike. Continuous pre-synaptic spikes were applied to the top electrode, which were insufficient to set the TiSbTe/SiC_0.11_:H artificial synapse for long-term memory retention; however, they were able to produce an excitatory post-synaptic current. As shown in Figure 6d, the strength of the artificial synapses was characterized by the SDDP index (An/A1 × 100%; *n* = 1, 2, 3..., 10). The increased duration of the spike was from 1 μs to 10 μs (Δt = 1 μs), and the amplitude of the spike was fixed at 1.2 V. In Figure 6d, A1 and A10 represent the currents of the device after the first and tenth spike, respectively. As shown in Figure 6c,d, the continuously increased spike duration caused a brief increase in synaptic weight. Unlike the short-term memory, the long-term memory (LTM) was able to change the synaptic weight permanently, which was the key element of the learning behavior. The analogue LTM of the Al/TiSbTe/a-SiC_0.11_:H/P^+^-Si device was evaluated by applying 20 positive pulses, followed by 20 negative pulses. As shown in Figure 6e, the conductance of the Al/TiSbTe/a-SiC_0.11_:H/P^+^-Si device increased/decreased when the continued positive/negative spikes were applied. The conductance updating was continued for five sweeping circles, exhibiting stable analogue switching behavior. As the representative characteristic of long-term memory, spike-timing-dependent plasticity (STDP) obeys the asymmetric Hebbian learning rule, which can reflect the connection between two neurons by modulating the relative timing (Δt) of activations from the pre-synapse and post-synapse. As shown in Figure 6a, the top electrode acted as the pre-synaptic terminal, and the bottom electrode was the post-synaptic terminal. The designed spike conditions are displayed in the inset of Figure 6f. The pre-synaptic spike (+) preceded the post-synaptic spike (−) when Δt > 0. The pre-synaptic spike (−) lagged behind the post-synaptic spike (+) when Δt < 0. The relationship between the synaptic strength and the time difference is shown in Figure 6f. In accordance with the learning rule, when the pre-spike preceded/lagged behind the post-spike, the connection strength of the synapse between the two neurons was reinforced/weakened. It can be seen that a larger conductance change was caused by a closer spike timing, which was consistent with the Hebbian learning rule. In order to visualize the information handling in the TiSbTe/a-SiC_0.11_:H neural network, the 6 × 6 synaptic arrays based on the Al/TiSbTe/a-SiC_0.11_:H/P^+^-Si memory were applied to image memorization by simulation, as shown in Figure 6g. The conductance was represented by the color level. In the initial state, the conductance of all synapses was distributed randomly. After the stimulation of 10 continuous pulses with a duration of 1 μs and an amplitude of 1 V, the conductance of the devices around the outside of the synaptic arrays was enhanced. As the number of pulses increased to 30, the clearness of the simulated image reached a high level.

## 4. Conclusions

In summary, ultra-high uniformity was obtained from the Al/TST/a-SiC_0.11_:H/P^+^-Si memristor by introducing a TST layer between the a-SiC_0.11_:H and the top electrode. Compared with that of the a-SiC_0.11_:H memristor, the cycle-to-cycle coefficient of variation in the high resistance state and the low resistance state of the TiSbTe/a-SiC_0.11_:H memristor was reduced by 92.5% and 66.4%. Moreover, the device-to-device coefficient of variation in the high resistance state and the low resistance state of the TiSbTe/a-SiC_0.11_:H memristor was decreased by 93.6% and 86.3%. HRTEM revealed that the TST nanocrystalline was formed in the TST layer during the first DC sweeping process, and remained in a crystalline state permanently in the following switching process. The localized electric field of the TST nanocrystalline was beneficial to confining the position of the Si dangling bond filaments in the a-SiC_0.11_:H film, which contributed to improving the uniformity of the device. The temperature-dependent I-V characteristic further confirmed that the bridge and rupture of the Si dangling bond nanopathway was responsible for the resistive switching of the Al/TST/a-SiC_0.11_:H/P^+^-Si device, which combined with the TST nanocrystalline to form the conductive pathway. The ultra-high uniformity of the TiSbTe/a-SiC_0.11_:H device ensured the successful implementation of biosynaptic functions such as spike-duration-dependent plasticity, long-term potentiation, long-term depression, and spike-timing-dependent plasticity. Furthermore, it proved possible to simulate visual learning capability by changing the conductance of the TiSbTe/a-SiC_0.11_:H device. Our discovery of the ultra-high uniformity of TiSbTe/a-SiC_0.11_:H memristor devices provides an avenue for their integration into the next generation of AI chips.

## Data Availability

Data can be available upon request from the authors.
